# Health and Cellular Impacts of Air Pollutants: From Cytoprotection to Cytotoxicity

**DOI:** 10.1155/2012/493894

**Published:** 2012-04-09

**Authors:** Karine Andreau, Melanie Leroux, Aida Bouharrour

**Affiliations:** Laboratory of Molecular and Cellular Responses to Xenobiotics, Unit of Functional and Adaptive Biology (BFA), CNRS EAC 4413, University of Paris Diderot, Sorbonne Paris Cité, 75013 Paris, France

## Abstract

Air pollution as one of the ravages of our modern societies is primarily linked to urban centers, industrial activities, or road traffic. These atmospheric pollutants have been incriminated in deleterious health effects by numerous epidemiological and *in vitro* studies. Environmental air pollutants are a heterogeneous mixture of particles suspended into a liquid and gaseous phase which trigger the disruption of redox homeostasis—known under the term of cellular oxidative stress—in relation with the establishment of inflammation and cell death *via* necrosis, apoptosis, or autophagy. Activation or repression of the apoptotic process as an adaptative response to xenobiotics might lead to either acute or chronic toxicity. The purpose of this paper is to highlight the central role of oxidative stress induced by air pollutants and to focus on the subsequent cellular impacts ranging from cytoprotection to cytotoxicity by decreasing or stimulating apoptosis, respectively.

## 1. Introduction

The air is fundamental and essential for living beings but epidemiological studies provide evidences of the harmful impacts of air pollution by increased cardiopulmonary morbidity and mortality as well as reproductive disorders and cancers [1, 2]. Some air toxics are released from natural sources but most are originated from anthropogenic sources, such as road traffic, construction, industrial, and agricultural activities [3]. Among almost two hundred hazardous air pollutants—mainly corresponding to suspended particulate matter and gases—only six are monitored by the Environmental Protection Agency (EPA) which sets the National Ambient Air Quality Standards (NAAQSs) for air particles, ozone, carbon monoxide, sulfur oxides, nitrogen oxides, and lead (Table 1). In addition, some other air pollutants are subjected to a specific attention because of their deleterious health impacts, like asbestos, mercury, chlorofluorocarbons, and polycyclic aromatic hydrocarbons (PAHs) [4].

At the present time, air pollution is considered as a major inducer of harmful health effects, especially due to solids or liquid droplets suspended in air and termed particulate matter (PM). PMs are usually defined as PM_10_, PM_2.5_ and PM_0.1_ that correspond to airborne particles with an aerodynamic diameter equal or less than 10, 2.5 and 0.1 microns, respectively. PM_10_ and PM_2.5_ are often classified as the “coarse” fraction; PM_2.5_–PM_0.1_ as the “fine” fraction of particles (FP) and PM_0.1_ correspond to the “ultrafine” fraction of particles (UFP). Although the transition from gasoline to diesel fuel has reduced emissions of carbon monoxide (CO), this has revealed new problems related to the emission of FP, UFP, and diesel exhaust particles (DEP). Engineered nanoparticles (NP), defined as particles having at least one dimension less than 100 nm, are in the same scale in size than atmospheric UFP, but NPs possess specific properties regarding their atomic scale capable of interacting directly with biological molecules. The surface parameter of PM is essential for understanding the biological effects of particulate pollution because size decrease is inversely correlated with the percentage of molecules on the surface and therefore with the surface reactivity. Thus, UFP and NP have a high surface reactivity that is responsible for the production of free radicals, for instance [5].

The chemical composition of PM varies according to environmental parameters (weather, continental, and/or regional influences) as well as to size. Indeed, PM_10_ are fragments from other larger particles observed during localized pollution episodes in urban areas and that may also include some pollen, spore, and plant. PM_10_ come from several sources like road transport, industrial, or construction activities [6] (Table 1). The urban aerosol mainly contains fine and ultrafine particles which consist of a core of elemental carbon from fossil fuel combustion and termed soots. Some inorganic components (ammonium, chloride, sulfates, nitrates, and metals), organic compounds—such as alkan, alkanoic acid, aliphatic acid, quinone, and PAHs—and biological species are adsorbed onto this carbon core [7].

The size of particles is directly linked to their deleterious potential on health. Indeed, FP, UFP, and DEP are inhaled with the air, penetrate deeply into the respiratory tract, and are mainly deposited in tracheobronchial and alveolar regions [5]. Industrial air pollution was clearly related to increased mortality and morbidity from respiratory and cardiovascular origin during episodes of heavily polluted fog (smog) in London in 1952 and the Ruhr in 1985 associated with 4,000 to 120,000 premature deaths, respectively, and an 8% increase in daily mortality. In addition, exposure to PM_2.5_, PM_10_, SO_2_, and black smoke has been shown as being responsible for asthma exacerbation in both adults and children [8]. The International Agency for Research on Cancer (IARC) classifies DEP as a possible carcinogen (Group 2A) [9]. Thus, epidemiological studies show that occupational exposure of truck drivers is associated with an increased incidence of lung cancer [10]. Based on the statistical model of an American study, the French Agency of Environmental and Occupational Health Safety (AFSSET) estimated in 2002 that 1117 lung cancer deaths were caused by PM_2.5_ exposure, a fraction of 11% [11]. Unlike the short-term effects linked to an inflammatory response, impacts of PM on carcinogenesis come from prolonged exposure. When mucociliary and alveolar clearance functions are exceeded, PM persist into lungs, leading to thickening of bronchial walls, an airway remodeling characterized by the hyperplasia of goblet and smooth muscle cells and a subepithelial fibrosis, as has been demonstrated in asthma and COPD (Chronic Obstructive Pulmonary Disease) [12]. Thereby, PM may act directly on the respiratory epithelium causing a range of various deteriorations to the total desquamation. To overcome this, the self-renewal of stem cells is accelerated, but proliferation and differentiation processes may escape to control and these adult lung stem cells are now considered as lung tumor initiators (see for review [13]). Given that the modulation of apoptotic cell death is an essential step in tumor initiation and promotion, this paper will focus on the molecular mechanisms of both induction and resistance to apoptosis with a particular attention to mitochondria, the main executor (or executioner) of apoptosis.

## 2. Apoptosis

 Since the remarkable scientific advances on the knowledge of cell death process, necrosis is not anymore considered as the only consequence of exposure to the toxic air pollutants. Indeed, impacts on activation or repression of apoptosis are now better described [17] and numerous *in vitro* studies demonstrated the modulation of apoptosis by environmental air pollutants including heavy or transition metals [18–20], carbon monoxide [21], and nondioxin-like PCBs (polychlorinated biphenyls) [22]. Apoptosis is a programmed cell death defined by morphological alterations (for review see [23]) leading to the progressive condensation of the cell into apoptotic bodies containing organelles or cytoplasmic fragments that are rapidly recognized and engulfed by neighboring cells and macrophages. The late-morphological changes are accompanied by biological alterations, such as the modification of lipid composition of the plasma membrane and permeability [24, 25], or the activation of various enzymes (e.g., phospholipase A2, DNase II, the caspase-activated DNase (CAD) [26], endonuclease G (EndoG) [27], the apoptosis-inducing factor (AIF) [28, 29]) leading to DNA fragmentation. All these apoptotic features may require activation of specific proteases called Caspases—for “cysteinyl aspartate-cleaving protease”—a family of proteins containing at least fourteen members in mammalians, eight of them actively participate to the execution of apoptosis while the others are involved in inflammation [30]. Under normal conditions, initiator Caspases-2, -8, -9, and -10 are expressed as inactive zymogens with a large N-terminal prodomain required for their autocatalytic activation into a tetrameric enzyme. When activated, the initiator caspase activates some executioner Caspases-3, -6, and -7, which in turn cleave specific substrates thus modifying multiple cellular functions such as DNA repair (DNA-PK, U1–70 kD, PARP), chromatin condensation (inhibitor of Caspase-Activated DNAse), or cytoskeleton stability (*α*-Fodrin, Lamin A, Actin); for review see [31].

The apoptotic cell death and caspases activation are mainly elicited by extrinsic and intrinsic pathways which are initiated by death receptors and intracellular events leading to mitochondrial dysfunction, respectively. In the extrinsic pathway, ligands of death receptors belonging to “tumour necrosis factor receptor” family (TNFR) promote formation of the multimolecular complex termed DISC (death-inducing signalling complex) [32] through the recruitment of adaptor proteins FADD (Fas-associated protein with death domain) and/or TRADD (TNF-receptor-associated death domain protein) and the subsequent activation of Caspase-8 and executive caspases.

In the intrinsic pathway (also called “mitochondrial pathway”), stimuli from different intracellular pathways (e.g., withdrawal of growth factors, exposure to toxins, hypoxia, bacterial or viral infections, physical or chemical stressors) converge on mitochondrial alterations that are the point of no return in apoptotic cell death. As reviewed elsewhere, permeabilization of outer and inner mitochondrial membranes (MMP) constitutes the limited and controlled step of the executive phase of apoptosis [33, 34]. In healthy cells, inner membrane (IM) is impermeable to protons in order to maintain the H^+^ gradient necessary to oxidative phosphorylations (OXPHOS) and mitochondrial transmembrane potential (ΔΨm). In dying cells, the IM's permeability increases to solutes less than 1.5 kDa and leads to the permeability transition caused by the opening of permeability transition pore complex (PTP) [35]. Despite a lack of consensus on the exact composition of the PTP, several proteins directly or indirectly constitute this complex such as the outer membrane- (OM-) inserted voltage-dependent anion channel (VDAC), the IM-located ANT (adenine nucleotide translocator), and the cyclophilin D in the mitochondrial matrix [36]. There is four isoforms of ANT (ANT 1–4) and three isoforms of VDAC (VDAC 1–3) that have antagonist effects on apoptosis, since VDAC1, ANT1, and ANT3 are proapoptotic proteins, while VDAC2, ANT2, and ANT4 are able to protect cells from death. In addition, several regulators interact closely with the PTP core proteins, in particular Hexokinase II, the translocator protein (TSPO), and Creatine Kinase which interact with VDAC in cytosol, outer membrane, and intermembrane space (IMS), respectively [35, 37, 38]. PTP opening can also be modulated *via* chemical modifications of PTP partners or through interaction with several pro- or antiapoptotic proteins. Indeed, thiols oxidation of ANT protein or proapoptotic members of the Bcl-2 family (i.e., Bax, Bak, and t-Bid) are well-known inducers of MMP [39, 40]. Generally, PTP opening and subsequent MMP results in ΔΨm dissipation associated with superoxide anion's production, swelling of the mitochondrial matrix as a consequence of the massive entry of water and solutes, and the release of many proapoptotic proteins from the IMS to the cytoplasm including Cytochrome *c*, Smac/DIABLO, and Omi/HtrA2 which participate to the caspase-dependent apoptotic pathway [41, 42]. Released Cytochrome *c* participates to the formation of a multiprotein complex termed “apoptosome” *via* physical interaction with the adaptor molecule Apaf-1 (apoptosis protease activating factor 1), the executioner procaspase-9, and ATP/dATP [43–46]. Furthermore, a caspase-independent apoptotic pathway can be activated by mitochondrial proteins AIF and EndoG that migrate towards nucleus to perform DNA fragmentation [33].

This mitochondrial central step may be positively or negatively modulated by Bcl-2 family proteins which mainly act through regulation of OM's permeabilization and Cytochrome *c* release. The Bcl-2 family contains three groups corresponding to prosurvival proteins (Bcl-2, Bcl-x_L_, Bcl-w, Mcl-1, and A1/Bfl1), proapoptotic effectors (Bax, Bak), and a third subfamily called BH3-only proteins (Bad, Bik, Hrk, Bid, Bim, Bmf, Noxa, and Puma) which modulates activation of both first groups. The upregulation of Bcl-2 or other antiapoptotic members and/or the downregulation of Bax/Bak proteins have been reported to impair MMP and apoptosis underlying their fundamental role in the state of life *versus* cell death [47]. Thereby, Strasser et al. proposed a new mechanistic model of how Bax and Bak could promote MMP, directly or indirectly; for review see [48]. Otherwise, the antiapoptotic proteins of the Bcl-2 family might inhibit cell death and MMP through two additional mechanisms: (i) the potential interaction with Apaf-1 protein leading to diminution of apoptosome formation or (ii) the direct inhibition of mitochondrial permeabilization. However the fact that Bcl-x_L_ might neutralize Apaf-1's function is still controversial, since data from different studies showing an inhibitory interaction between Bcl-x_L_, Caspase-9, and Apaf-1 [49, 50] were rapidly refuted [51, 52]. Bcl-2 and Bcl-x_L_ are related to some bacterial proteins and data demonstrated their ability to form pores into membranes, to prevent the proton efflux triggered by calcium or reactive oxygen species (ROS) and the maintenance of mitochondrial ADP/ATP exchange [53, 54]. Bcl-2 also prevents excessive ROS production and impacts of the subsequent oxidative stress [55].

As a source and a cellular target of ROS, mitochondria regulate glucose metabolism, differentiation, or cell death and might play an important role in tumorigenesis. Currently, a new emerging concept considers mitochondrion as a ROS-signaling integrator [56, 57]. Mitochondrion is the site of OXPHOS by which ATP is formed by coupling with the electrons' transfer from a donor (NADH or FADH2) to the final acceptor oxygen of the mitochondrial respiratory chain. However, about 2% of the electrons escape from sites in complex I and/or complex III to react directly with oxygen thus generating superoxide anion (O_2_^∙−^) [56]. This leakage of electrons may appear in hypoxia conditions but could also participate under physiological conditions to various signaling pathways, since O_2_^∙−^, H_2_O_2_ (hydrogen peroxide) and HO^∙^ (hydroxyl radical) are considered as intracellular messengers. Mitochondria seem to be the most potent intracellular source of ROS since the mitochondrial matrix concentration of O_2_^∙−^ was estimated to be 5- to 10-fold higher than that in the cytosol [58]. The Mn-superoxide dismutase (SOD) localized in the mitochondrial matrix rapidly dismutes O_2_^∙−^ in H_2_O_2_ which in turn can be decomposed by Catalase or may interact with O_2_^∙−^ by the Haber-Weiss reaction, or with Fe^2+^ (or Cu^+^) by the Fenton reaction, leading to the generation of HO^∙^. The mitochondrial ROS generation can be responsible for activation of death pathways by inducing (i) nuclear and mitochondrial DNA damages (i.e., formation of 8-hydroxydeoxyguanosine) leading to p53-dependent cell death [59], (ii) activation of signaling pathways involving NF-*κ*B, JNK, or p38 MAPK [60, 61], (iii) MMP increase and Ca^2+^-induced PTP opening [39, 62], and (iv) Cytochrome *c* release by oxidation of the anionic phospholipid cardiolipin [63, 64]. Nevertheless, mitochondrial ROS can also participate to protection against apoptosis by activation of antioxidant systems such as GSH (L-*γ*-glutamyl-L-cysteinylglycine) and multiple GSH-linked antioxidant enzymes (i.e., Gluthatione Peroxidases 1 and 4 [65, 66], Glutaredoxin 2 [67, 68], Glutathione S-transferase *α* [69]).

## 3. Air Pollutants and Cytotoxic Cell Death

Apoptosis is one of the possible consequences of acute or chronic exposure to air pollutants and various toxicants—such as PM, metals, and pesticides—are capable to target mitochondria, directly or indirectly [70–72]. For instance, rotenone which is used as pesticide inhibits the mitochondrial complex I. In addition, other pesticides such as pentachlorophenol and 2,4-dinitrophenol (DNP) induce cytotoxicity by uncoupling ATP synthesis and the mitochondrial H^+^ gradient. Indeed, the small lipophilic molecule DNP captures and carries protons out of the IMS leading to the H^+^ accumulation into the matrix and the disruption of the pH/H^+^ gradient [73].

**Figure 1:**
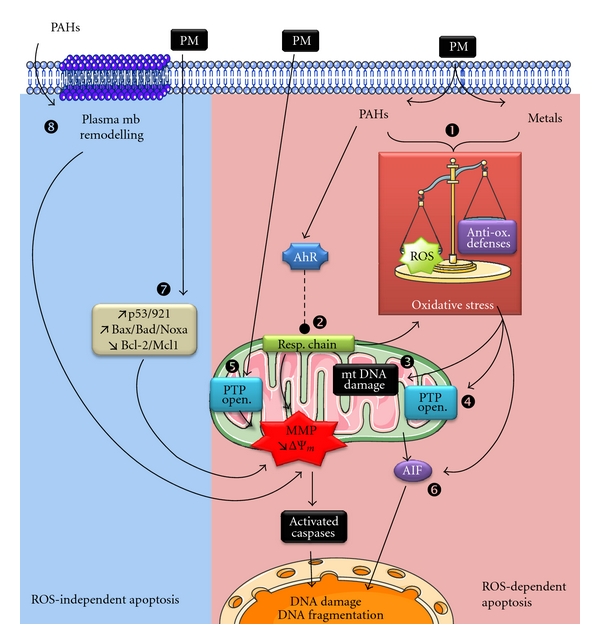
ROS-dependent and -independent apoptosis induced by particulate pollutants. Particulate matter (PM) or their compounds (PAHs and metals) provoke apoptotic cell death through ROS-dependent (pink zone) or ROS-independent (blue zone) pathways. Actually, particulate pollutants are considered as potent ROS generators from organic (i.e., PAHs) or metallic compounds (1) and leading to oxidative stress as the result of the unbalance between ROS production and activation of antioxidant defenses. Senft et al. demonstrated that AhR activation might regulate the mitochondrial respiratory chain function and induce production of O_2_^∙−^ and H_2_O_2_ from mitochondria ([83], (2)). As a consequence of oxidative stress, mitochondria are harmed by ROS that are responsible for damage of mitochondrial DNA (3), mitochondrial lipid peroxidation, and opening of PTP complex (PTP open. (4)). Mitochondrial membrane permeabilization (MMP) and PTP opening might also be a direct effect of diesel particles on isolated mitochondria ([84], (5)). As an additional pathway of the ROS-dependant apoptosis induced by air pollutants, the apoptogenic activity of AIF might be enhanced by xenobiotics, air pollutants, or their ROS derivatives (6). Some other ROS-independent signaling pathways have been identified such as the upregulation of proapoptotic proteins and/or the repression of prosurvival Bcl-2 family proteins (7). Recent publications also demonstrated a new mechanism of apoptosis triggered by PAHs through alterations of lipid rafts' composition and remodeling of the plasma membrane (8). Illustrations carried out thanks to Servier Medical Art.

Although many studies describe the ability of air pollutants to trigger some characteristic features of apoptosis, only few detailed mechanistic studies have been published, the majority focusing solely on the oxidative stress emergence as a result of the unbalance between ROS production and activation of antioxidant defenses. Indeed, publications dealing with the cytotoxic consequences of airborne particles showed an induction of apoptosis associated with cellular stress, ROS production [74], ΔΨm drop, caspases activation [75], and DNA fragmentation [70, 76, 77]. The cell death often demonstrated in experiments performed with PM in normal human lung tissue or airway epithelial cells was a mitochondria-mediated apoptosis, characterized by a marked reduction of mitochondrial dehydrogenase activity and the cytoprotective effects of mitochondrial inhibitors (e.g., rotenone, DIDS) [78, 79]. Cytochrome *c* release, activation of caspases-9 and -3, and PARP-1 cleavage were also observed after exposure to urban and industrial PM_2.5_ in correlation with the induction of an oxidative stress studied by formation of 8-hydroxy-2′-desoxyguanosine (8-OHdG) [78, 80]. Thus, short-term exposure studies performed in respiratory cells with high doses of PM or its components led to a consensus that health effects as well as cytotoxic impacts of particulate pollution mainly involve ROS production and oxidative stress (Figure 1) [81, 82].

Increased production of ROS was first clearly identified as the central step of the proinflammatory response (GM-CSF, IL-6, IL-8, TNF-*α*) induced upon exposure to air pollutants *via* ROS-sensitive transcription factors such as NF-*κ*B and AP-1 [82, 85]. Actually, particulate pollutants are considered as potent oxidants, and the induced intrinsic pathway of apoptosis may be associated with oxidative stress generated from organic (i.e., PAHs, nitro-PAHs/ketones/quinones) as well as inorganic compounds adsorbed on the surface of particles [84, 86–88]. Thus, PAHs induced-apoptosis is mainly mediated *via* the mitochondrial pathway (Caspase-3 activation, AIF, and EndoG release) in a p53-dependent manner in hepatic cells and macrophages [89–91], even if the causal relationship between genotoxic effects of BaP and induction of apoptosis is not established for all cell types [92]. Organic components are able to mimic the apoptogenic impact of PM in various cell types through activation of the aryl hydrocarbon receptor (AhR). AhR is a cytoplasmic ligand-dependent transcription factor which translocates to the nucleus in order to bind specific Xenobiotic Responsive Elements in target genes promoters, leading to activation of phase I and II metabolizing enzymes and contributing to detoxification. For instance, phase I enzymes such as cytochrome P450 oxidase 1A1 produce H_2_O_2_ by metabolizing benzo(a)pyrene (BaP) [93] into a reactive intermediate (anti-7,8-dihydrodiol-9,10-epoxy-benzo(a)pyrene, BPDE) [94] known to trigger DNA damage and carcinogenesis [95]. *In vivo *studies performed in mice exposed to TCDD (2,3,7,8-tetrachlorodibenzo-*p*-dioxin) showed an important oxidative response in correlation with the high affinity of the TCDD-AhR binding, suggesting a strong link between oxidative stress, Ah receptor, and its target genes CYP1A1 and 1A2 [96].

In this context, Senft et al. were interested to understand the involvement of AhR in mechanisms responsible for the dioxin-induced mitochondrial oxidative stress. These authors reported that liver mitochondria from mice exposed to TCDD (15 mg/kg) during three consecutive days showed a decrease of aconitase activity as a marker of mitochondrial ROS production, a mitochondrial generation of O_2_^∙−^ and H_2_O_2_ in the presence of succinate, as well as an activation of glutathione peroxidase-1 and glutathione reductases [83]. Interestingly, AhR knockout mice are protected from TCDD-induced ATP depletion, production of ROS, and the resultant oxidative stress response, suggesting that AhR might regulate the activity of mitochondrial electron transport chain, especially the complexes III (stimulation) and IV (inhibition), and that the major source of the direct AhR-mediated oxidative stress response has a mitochondrial origin.

Furthermore, mitochondrion may be a site of ROS generation also in response to metallic environmental pollutants, as shown with cadmium or hexavalent chromium that trigger ROS production from the electron transfer chain as well as from the NADPH oxidase activity [97, 98]. In addition to organic compounds, heavy and transition metals such as vanadium, cadmium, mercury, lead, aluminum, titanium, chromium, iron, cobalt, nickel, copper, and zinc are often found in atmospheric pollutants and measured as adsorbed inorganic compounds on FP and UFP. Metals affect human health, especially when these toxicants compete with essential elements and thus modify many cellular processes [99]. Some metals are janus elements since they are both prominent inducers of ROS and essential cofactors for some antioxidant enzymes. As reviewed by Pulido and Parrish, transition metals promote apoptosis through ROS generation, mitochondria dysfunction, activation of MAPK, p53 and caspases, or downregulation of antiapoptotic proteins of Bcl-2 family [100]. Metals and the water-soluble fractions of PM are also known to cause inflammation and cancer mostly due to DNA damage as a consequence of ROS generation by Fenton reaction (see Section 2). Indeed, carcinogenic metals (i.e., As, Cd, Cr, Ni) promote apoptosis with DNA-base modifications, strand breaks and rearrangements [101]. Generation of ROS, decrease of intracellular GSH, accumulation of Ca^2+^, loss of ΔΨm, upregulation of Caspase-3, downregulation of Bcl-2, and deficiency of p53 protein led to arsenic-induced apoptosis [102, 103]. In case of cadmium, metallothionein expression determines the cell death fate (between apoptosis and necrosis), but Cd-induced apoptosis is due to inhibition of antioxidant enzymes, mitochondrial dysfunction [104], and PTP opening, probably through its interaction with thiol groups of ANT [105], whereas ROS and p53 contribute to apoptosis caused by chromium and selenium [106–108]. In addition, PM containing high levels of noncarcinogenic metals (i.e., cobalt, lead, iron, and zinc) were often shown to provoke ROS production (e.g., H_2_O_2_) leading to apoptosis through the mitochondrial pathway [109–113]. Under apoptotic conditions, zinc is also able to increase p53 expression and function probably by stabilizing this protein which contains a tightly bound zinc atom necessary for its DNA binding activity [114, 115].

As a consequence of oxidative stress, mitochondria are often damaged by ROS and experiments performed on HepG2 cells and liposomes clearly showed that O_2_^∙−^ alone seems to elicit apoptosis and a rapid and massive release of Cytochrome *c* independently of PTP opening but rather through a VDAC-dependent permeabilization of the outer mitochondrial membrane [116]. These results are in contradiction with those obtained by Xia et al. which performed, to the best of our knowledge, the only study of the prospective direct effect of Diesel particles on mouse liver-isolated mitochondria [84]. Xia et al. showed that the aromatic fraction of DEP can directly induce mitochondria swelling and depolarization leading to calcium overload in matrix [84]; this might be related to the massive decrease of the content in cardiolipin published earlier [117]. As mitochondrial swelling can be due to the long-lasting opening of PTP or the closure of VDAC, Xia et al. assessed the effect of the PTP inhibitor cyclosporine A (CsA) and demonstrated its ability to counteract DEP-induced swelling, suggesting that these organic compounds may directly promote PTP-mediated MMP [84]. In addition, oxidation of cardiolipin may be a crucial event of cell death triggered by xenobiotics as demonstrated by *in vivo* inhalation exposure to single-walled carbon nanotubes and *in vitro* experiments of LPS-induced apoptosis on pulmonary artery endothelial cells. Indeed, cardiolipin hydroperoxides and their hydroxy-derivatives are prominently accumulated in inflammatory, apoptotic, and oxidative stress conditions [118]. However, mitochondrial lipid peroxidation is not the only oxidative stress consequence responsible for mitochondria-driven apoptosis since O_2_^∙−^ and H_2_O_2_ can damage proteins as well as mitochondrial DNA. Mitochondrial DNA (mtDNA) is one of the main targets of mitochondrial ROS due to the close proximity of site production and the lack of protective histones. TCDD at low doses was shown to induce preferentially mitochondrial *versus* nuclear genotoxicity as assessed by 8-OHdG, reduction in mtDNA number, and increase in mtDNA deletions [119, 120].

As an additional pathway of the ROS-dependent apoptosis induced by air pollutants, the apoptogenic activity of AIF is controlled by the redox status as oxidized monomers have higher DNA affinity than NADH-reduced dimers [121]. Mitochondrial AIF is a NADH-dependent oxidoreductase containing a flavin adenine dinucleotide (FAD) and which is tethered to the inner membrane of mitochondria and participates to the caspase-independent pathway of apoptosis. However, a recent published data demonstrated that AIF has a quinone reductase activity that is around thousand-fold lower than Cytochrome P450 or NADPH : quinone oxidoreductase (NQO1) normally involved in the phases I and II of the xenobiotic detoxification processes [122]. This suggests that interaction of AIF with xenobiotics, air pollutants, or their ROS derivatives might promote its oxidized form and enhance its apoptogenic activity. Thereby, apoptosis induced by PM or wood smoke extracts on human alveolar macrophages and pulmonary artery endothelial cells was associated with AIF upregulation and its translocation to nucleus [123, 124]. In addition to the mitochondrial pathway, different studies also demonstrated that the extrinsic pathway of apoptosis (with TNF-*α* secretion, caspases-8 and -3 activation) is involved in the cytotoxic impacts of FP, UFP, and NP [125–127]. Data published in 2006 showed that apoptosis induced by fine particles in the lung epithelial cells has taken place in parallel with the induction of proliferation and that the two antagonistic phenomena appear to be induced by oxidative stress or EGFR [127, 128]. Finally, posttranslational oxidative modifications of proteins (i.e., nitrosylation, hydroxylation, glutathionylation of cystein residues) have been shown to promote aberrant activation of signal transduction cascades. Among these pathways, modulation of kinases ASK/JNK, Akt, or MAPK, or transcription factors Nrf2, NF*κ*B, AP-1, and p53 was clearly demonstrated following exposure to various environmental pollutants whose cellular effects are establishment of both an inflammatory and an apoptotic response [80, 129–132].

Through all examples cited previously, it is clear that cytotoxicity of air pollutants (PM, PAHs, metals, or herbicides) in various cells [70, 78, 133] mainly incriminates the excessive production of ROS capable of targeting mitochondria by different mechanisms (Figure 1) [83, 134]. At the same time as the ROS-dependent apoptosis, some other oxidative stress-independent signaling pathways have been identified. For instance, different PM were shown to upregulate the expression of potent regulators of the mitochondrial checkpoint such as p53 and its targets p21, Noxa, Bax, Bad, and Bim in parallel to the repression of Bcl-2 and Mcl-1 [70, 78, 125, 135]. Moreover, recent publications also demonstrated a new mechanism of apoptosis triggered by 1-Nitropyrene and BaP through lipid accumulation [136], alterations of plasma membrane microstructures, especially modulation of the Na^+^/H^+^ exchanger, inhibition of the gap junctional intercellular communication [137], and alteration of lipid rafts' composition [138]. These interesting outcomes emphasize the possible dialogue between plasma membrane alterations and cell death. Impacts on the plasma membrane remodeling might provide additional mechanistic explanations of how some chemicals exert their carcinogenic effect [25].

## 4. Cytoprotective Effects of Air Pollutants

 Since mid-twentieth century, the incidence in lung cancer had rapidly increased and, in addition to cigarette smoke, indoor and outdoor air pollution was also questioned through epidemiological studies. The earliest studies underlined the higher incidence of lung cancer in urban *versus* rural areas, in relation to the nature or concentration of airborne particles. The IARC classified several air pollutants as human carcinogens (group 1, e.g., BaP, Chromium VI compounds, tobacco smoking), probably carcinogens (group 2A, e.g., dibenzo(a,h)anthracene, engine diesel exhaust, lead compounds), possibly carcinogens (group 2B, e.g., benzo(k)fluoranthene, gasoline exhaust, lead), not classifiable as to its carcinogenicity to humans (group 3, e.g., benzo(g,h,i)perylene, chromium III compounds), or probably not carcinogenic to humans (group 4) [9, 139, 140]. In particular, extracts of PM, BaP, and 1,6-dinitropyrene provoke DNA adducts, mutagenic effects in bacterial and mammalian cells, chromosomal damages, and cell transformation. Most of human cancers are carcinoma derived from epithelial cells, and in the case of lung cancers, the most common are squamous cell carcinoma (epidermoid carcinoma), large cell carcinoma, small cell carcinoma, and adenocarcinoma [141]. All cancerous cells have acquired six capabilities during the multistage process of tumorigenesis: (i) self-sufficiency in growth signals, (ii) insensitivity to growth-inhibitory signals leading to (iii) limitless replicative potential, (iv) evasion to apoptotic cell death, (v) sustained angiogenesis, and (vi) tissue invasion and metastasis [142]. During chronic exposure to air pollutants, acquisition of resistance towards apoptosis might be a significant step of the molecular mechanisms involved in the initiation and promotion of tumors as demonstrated by Teranishi et al. [143]. Unfortunately, too few toxicological studies have investigated this phenomenon of apoptosis resistance induced possibly by air pollutants and have tried to decipher in detail the underlying molecular mechanisms. In order to delineate mechanisms leading to lung cancers due to exposure to cooking oil fumes (COFs) and its main aldehyde component 2,4-decadienal (2,4-DDE), Hung et al. reported that these pollutants promote survival and proliferation of alveolar cancer cells A549 through increased expression of IAP1, IAP2, Survivin, and Cyclin D1 and the concomitant decrease of XIAP, Caspase-3, and p21 level [144].

 As described previously, particulate pollutants or their components may induce cytotoxicity following high-concentrations exposure. Ferecatu et al. initiated studies to determine the specific effect of low doses of airborne particles on different bronchial epithelial cells (tumoral, immortalized, and primary cells) regarding induction or reduction of the apoptotic process. The authors demonstrated that PM_2.5_ are not cytotoxic but rather trigger a resistance to mitochondrial apoptosis towards well-known cell death inducers (calcimycin (A23187), staurosporine, and oligomycin) [145]. The reduction of apoptosis observed after particle exposure is not related to the proinflammatory response and EGF pathway but is mediated by water-soluble as well as organic components such as heavy PAHs. Among all the water-soluble compounds of PM, a possible candidate responsible for the cytoprotective effect is zinc (Zn), already known to inhibit apoptosis and minimize the oxidative stress (e.g., lipid peroxidation) [146]. Zn may protect cells both directly by stabilizing lipids and proteins of cellular and organelles membranes and indirectly *via* the maintenance of glutathione levels [147]. The protective effect of Zn was also assigned to the reduction of DNA fragmentation, processing of procaspase-3 [148] and activation of cytoprotective signaling pathways (including Akt, ERK). In addition, some divalent transition metals (Mg^2+^, Sr^2+^, and Mn^2+^) can competitively inhibit the calcium-induced PTP opening through a still-unknown mechanism, suggesting a possible direct and protective effect of metallic compounds on mitochondria [149].

Several studies have reported the cytoprotective effects of organic compounds of PM such as PAHs [145, 150] or their metabolites [143, 151], TCDD [152], non-Dioxin-like PCBs [153], and DEP [154]. The exact mechanisms of apoptosis inhibition are not fully understood but *in vitro* studies have shown the necessity of protein synthesis [155], p53 modulation [153, 156], and AhR activation [145]. Indeed, exposure of human bronchial epithelial cells for 4 hours with different PAHs, prior to A23187-treatment, showed a marked resistance to mitochondria-driven apoptosis only with PAHs containing at least five-aromatic rings, which are the most toxic and potent inducers of receptor Ah (Table 2) [157]. The need of protein synthesis and AhR activation might be linked to the transcription factor function of AhR that could either induce antiapoptotic genes or inhibit proapoptotic ones. This assumption is based on results which have shown that AhR directly interacts with E2F1 leading to the reduction of E2F1-mediated proapoptotic genes such as *apaf-1* [158]. Moreover, the cytoprotective effect of AhR ligands is effective at the mitochondrial checkpoint of apoptosis by upregulating expression of antiapoptotic genes such as *bcl-2*, *bcl-* *x_L_*, *mcl-1*, *agr2*, or *vdac2 *[159–161].

Signaling pathways other than AhR were also found in the cytoprotective effects of air pollutants, especially protein kinases (e.g., Akt [162], ERK [163], JNK, PKA [164]), or the transcription factor Nrf2 (nuclear factor (erythroid-derived 2)-like 2). PI3K/Akt and AhR pathways seem to be interdependent for the cellular response to xenobiotics, since the presence of AhR is required for the cytoprotective function of Akt [165]. In addition, the antiapoptotic effect of DEP and BPDE (a BaP genotoxic metabolite) was linked to phosphorylation and activation of Akt [151], with the possible involvement of Thioredoxin-1 as demonstrated *in vivo* [166]. Furthermore, Akt promotes type II Hexokinase (HK II) phosphorylation and binding to the OM leading to the stabilization of PTP complex in closed conformation and the inhibition of Ca^2+^-induced Cytochrome *c* release. Akt-dependent phosphorylation of mitochondrial HK II is further favored when glycogen synthase kinase 3*β* (GSK3*β*) is inactivated by Akt phosphorylation [167]. Finally, activation of NF-*κ*B and AP-1 by exposure to airborne PM or cigarette smoke is well documented and admitted as being part of inflammatory or proliferative response [168, 169], but their role in the modulation of apoptotic cell death remains unclear.

As recently reviewed, air pollutants are also inductors of Nrf2 which regulates the expression of phase II detoxifying enzymes as well as cytoprotective antioxidants [170]. Under normal conditions, Nrf2 is a cytoplasmic protein linked to its repressor Keap1 (Kelch-like ECH-associated protein) but exposure to low levels of electrophiles and ROS causes the nuclear translocation of Nrf2 and the subsequent expression of target genes containing antioxidant response elements (ARE). Indeed, numerous published studies have reported activation of Nfr2 after exposure of murine macrophages or human bronchial epithelial cells to DEP, PAHs, and UFP [81, 171, 172]. Among the antiapoptotic target proteins of Nfr2, two antioxidant enzymes are of particular interest because of their location and action on the mitochondria: Heme Oxygenase-1 (HO-1) and Glutathione S-transferase (GST) isoenzymes. Expression and activity of HO-1 are dramatically increased in mitochondrial fractions of human alveolar and bronchial cells exposed to cigarette smoke extract as an attempt to counteract its toxic effects, since the overexpression of HO-1 inhibits cell death and maintains ATP levels [173]. The cytoprotective mechanism of HO-1 involves its enzymatic reaction products such as biliverdin, carbon monoxide (CO), and ferrous iron [174]. In response to Fe^2+^ production, ferritin protein stability is increased and may protect cells from oxidative and Fas-induced apoptosis. However, biliverdin and free iron do not have any protective activity against oxidative stress-induced hepatic apoptosis, suggesting that CO may be the key molecule [175]. Indeed, CO protects against oxidative injuries and cell death, since in addition to limiting the translocation of Bcl-2 family proteins to mitochondria and the Cytochrome *c* release, Queiroga et al. proposed a new cytoprotective effect of CO directly on mitochondria [176]. They observed that a fifteen-minutes pretreatment with low doses of CO was able to prevent the calcium-induced swelling and depolarization of liver isolated mitochondria. Otherwise, GST enzymes are involved in detoxification of endogenous toxic metabolites, superoxide radicals, and xenobiotics. Cytosolic and mitochondrial GST (*α*, *μ*, *π*, and *θ*) were shown to be upregulated by numerous xenobiotics and some AhR ligands (TCDD, *β*-naphthoflavone) [177]. However, effect of mitochondrial GST on the direct control of this organelle is still subject of controversy: on the one hand, these enzymes are assumed to prevent cardiolipin oxidation and MMP [178], while on the other hand, Aniya's team demonstrated that the mitochondrial membrane-bound GST1 is activated by S-glutathionylation and contributes to Cytochrome *c* release through PTP opening [179].

ROS are possibly involved in tumor progression, metastasis, and multiple signaling pathways elicited by atmospheric xenobiotics. The correlation of intracellular oxidation—due to light-modified derivatives of BaP (BaP-1,6-dione, BaP-3,6-dione, BaP-4,5-dihydrodiol and 2-hydroxy-BaP-1,6-dione)—with the protection against serum withdrawal-induced apoptosis has clearly suggested that a certain dose of ROS enhances cell proliferation and survival [143]. Under physiological conditions, cell viability is critically dependent on the maintenance of functional antioxidant systems especially in lung cells which are regularly exposed to air pollutants. GSH is one of the most abundant antioxidant found in the extracellular epithelial lining fluid and into respiratory cells from trachea to alveoli [181]. According to this preponderant role of GSH, administration of antioxidants, such as N-acetylcysteine (NAC), effectively prevents air pollutant-induced apoptosis [126, 171, 182] and *in vivo *exposure to dioxin increases levels of mitochondrial glutathione in wt, *cyp1a1* ^−/−^ and *cyp1a2* ^−/−^ mice, but not in *ahr* ^−/−^ mice [83].

## 5. Conclusion

**Figure 2:**
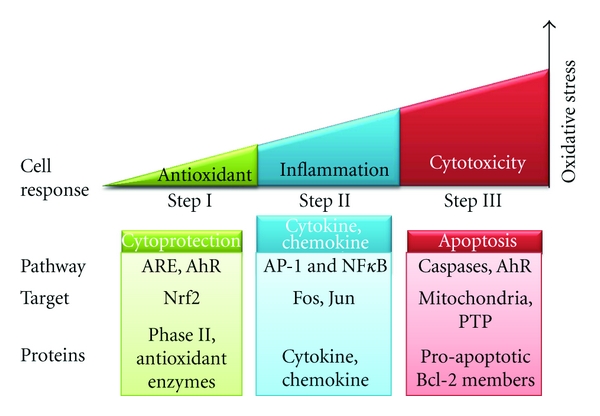
Hierarchical oxidative stress model in response to air pollutants. Low ROS production (Step I) induces activation of cellular antioxidant systems to restore redox homeostasis. If this protection is insufficient, the increased stress (Step II) triggers an inflammatory response through MAPK and NF*κ*B pathways. At a final stage (Step III), all the defense systems are overwhelmed and high ROS levels lead to PTP opening and cell death by apoptosis. These responses depend on the pro/antioxidant balance which varies from one living being to another (adapted from [180]).

In conclusion, impacts of air pollutants are mainly related to the redox status and the mitochondrial function of target cells. As described in this paper, proteins and signaling pathways involved in both cytoprotective and cytotoxic effects converge to the pro/antioxidant balance which determines the cellular response to environmental aggressions according to the hierarchical model proposed by Li et al. [180] (see Figure 2). Exposure to environmental pollutants may result in cellular disorders responsible for tissue damage and is therefore perceived as a cellular stress. A minor stress will induce a cellular response characterized by metabolic, morphological, or signaling alterations in order to deal with it. This phenomenon, termed adaptation, involves several processes such as hypertrophy, hyperplasia, or atrophy of the cells. Persistent exposures may also result in metaplasia (replacement of one by another cell type as is the case for the ciliated columnar cells that are replaced by squamous cells in the cigarette smokers' epithelium). In case of severe injuries, adaptative processes are overwhelmed leading to cell death by necrosis, apoptosis, or autophagy. Air pollutants can directly affect the respiratory epithelium and cause transient damages (i.e., loss of cilia and tight junctions) until total desquamation. Thus, cellular adaptation allows tissue remodeling required for the repair/regeneration of the damaged lung epithelium.

In this context, the cytoprotective effects demonstrated for some xenobiotics might reflect the setting up of an adaptative mechanism converging to the inhibition of cell death deleterious for the lung tissue. However, as it was well described by Barouki [183], some adaptative mechanisms necessary to inhibit direct toxicity may have side effects that accumulate during repeated exposures. A plausible assumption for the exacerbation of lung cancers would be that resistance to apoptosis might occur into self-renewing stem cells known to be involved in physiological regeneration of the epithelium and suspected of being initiators of lung tumors [13].

 Despite the latest significant advances, the specific mechanisms responsible for cytotoxic effects of air pollutants remain to be deciphered in more detail, mostly on the issues of chronic as well as multiple exposures to low concentrations of pollutants. Moreover, understanding the adaptive cytoprotective process is an important issue that should be considered in the risk assessment of air pollution.

## Figures and Tables

**Table 1:** Air pollutants. The National Ambient Air Quality Standards (NAAQSs) are set by the Environmental Protection Agency under authority of the Clean Air Act and define the maximum allowable concentrations of outdoor air pollutants in the USA. Units of NAAQS are parts per million (ppm) by volume, parts per billion (ppb-1 part in 1,000,000,000) by volume, milligrams per cubic meter of air (mg/m^3^), and micrograms per cubic meter of air (*μ*g/m^3^). Average time refers to time for which the values of NAAQS should not be exceeded in the ambient air [14–16].

Pollutants	Main sources	NAAQS	
		Level	Average time
Asbestos	Electrical and building insulation (fiber cement)		

Carbon dioxide (CO_2_)	Fossil fuel combustion		

Carbon monoxide (CO)	Incomplete combustion, exhaust from motor vehicles, emissions from certain industrial processes (agglomeration of ore, steel, waste incineration)	35 ppm (10 mg/m^3^) 9 ppm (10 mg/m^3^)	1 hour 8 hours

Chlorofluorocarbons (CFCs)	Use in consumer goods (aerosol propellants, foams, fire extinguishers, refrigerants)		

*Dioxins and dioxin-like compounds *	Byproducts of various industrial processes		
Polychlorinated dibenzo-p-dioxins (PCDDs)	Waste incineration, metal smelting and refining, chlorinated pesticides, and herbicides		
Polychlorinated dibenzofurans (PCDFs)	Environmental accidents contamination, waste incineration, chlorinated pesticides, and herbicides		
Polychlorinated biphenyls (PCBs)	Used as coolants and insulating fluids for transformers and capacitors, and as plasticizers in paints and cements, additives in flexible PVC coatings		

Hydrogen sulfide (H_2_S)	Paper pulp production and oil refineries		

Methane (CH_4_)	Coal mines exploitation, garbage landfills, livestock, gas distribution		

Nitrogen dioxide (NO_2_)		100 ppb 53 ppb	1 hour Annual

Nitrogen monoxide (NO)			

*Nitrogen oxides (NOx)*	Fossil fuel combustion, industrial processes (nitric acid production, fertilizer manufacturing, surface treatment)		

Nitrous oxide (N_2_O)	Fossil fuel combustion, some industrial processes, motor vehicles, soils, and oceans		

Ozone (O_3_)	Tropospheric ozone formed from reaction between UV, NOx, and VOC	0.12 ppm (235 *μ*g/m³) 0.075 ppm (150 *μ*g/m³)	1 hour 8 hours

*Particulate matter (PM)*			
PM_10_	Natural dust, sea salt, industrial, agriculture, and forestry activities	150 *μ*g/m^3^	24 hours
PM_2.5_	Fossil fuel combustion, road traffic, and other transports, agriculture, and manufacturing	35 *μ*g/m^3^ 15.0 *μ*g/m^3^	24 hours Annual
PM_0.1_	Residential heating, road transport, manufacturing, agriculture, waste processing plants		
Polycyclic aromatic hydrocarbons (PAHs)	Incomplete combustion of organic material (wood burning, fossil fuel combustion, etc.)		
Sulfur dioxide (SO_2_)	Sulfur-containing fossil fuel combustion (coal, lignite, petroleum coke, heavy fuel oil, heating oil, diesel),	75 ppb	1 hour
		0.14 ppm (365 *μ*g/m^3^)	24 hours
		0.030 ppm (80 *μ*g/m³)	Annual
*Toxic metals *			
Antimony (Sb)	miscellaneous plastics manufacturing, petroleum products, and fabricated structural metal products, thermal power generation		
Arsenic (As)	Heavy fuel oil combustion		
Cadmium (Cd)	Waste incineration, heavy fuel oil, and biomass burning		
Chromium (Cr)	Production of glass, cement, ferrous metallurgy, and foundries		
Cobalt (Co)	Nuclear facilities, production of steel and alloys		
Copper (Cu)	Combustion and waste treatment, processes of ferrous and nonferrous metallurgy		
Lead (Pb)	Road transport, electric batteries production	0.15 *μ*g/m^3^	Rolling 3 Months
Mercury (Hg)	Coal and oil burning, chlorine production, incineration of household, and industrial waste
Nickel (Ni)	Heavy fuel oil combustion
Selenium (Se)	Glass production, heavy fuel oil combustion
Vanadium (V)	Oil refineries, combustion of fossil fuels
Zinc (Zn)	Coal and heavy fuel combustion, ferrous and nonferrous metallurgy, waste incineration

Volatile organic compounds (VOCs)	Road transport, industrial processes involving the use (basic and fine chemicals, metal degreasing, paint application, printing, adhesives, rubber, etc.), or not of solvents (petroleum refining, use of CFCs, production of alcoholic beverages), household products		

**Table 2:** The cytoprotective effect of PM_2.5_ is related to PAHs with five-aromatics rings. Epithelial 16 HBE cells were pretreated during 4 hours with phenanthrene (124 nM), fluoranthene (268 nM), benzo(b)fluoranthene (333 nM), benzo(k)fluoranthene (333 nM), benzo(a)pyrene (270 nM), dibenzo(a,h)anthracene (35 nM), benzo(g,h,i)perylene (443 nM), and indeno(1,2,3-cd)pyrene (217 nM) prior to induction of apoptosis by A23187 (3 *μ*M) for 20 supplementary hours. Results are mean ± SD (*n* = 6). Significance was calculated with Dunnett's test (*P < 0.01 versus vehicule cyclohexane 1%). Percentages of DiOC low and PI high refer to cells showing either a drop of ΔΨm or a permeabilization of the plasma membrane measured using DiOC and propidium iodide (PI) probes, respectively. Note that a 4 h vehicle pretreatment provides 93.00 ± 3.31% DiOC low and 96.97 ± 7.18% PI high of A23-induced apoptosis, respectively. Moreover, a 4 h PM_2.5_ exposure (10 *μ*g/cm²) provides 64.33 ± 9.89% DiOC low and 39.45 ± 8.50% PI high of A23-induced apoptosis, respectively. The relative toxic potency of individual PAH compared to BaP is given as the toxic equivalency factor (TEF).

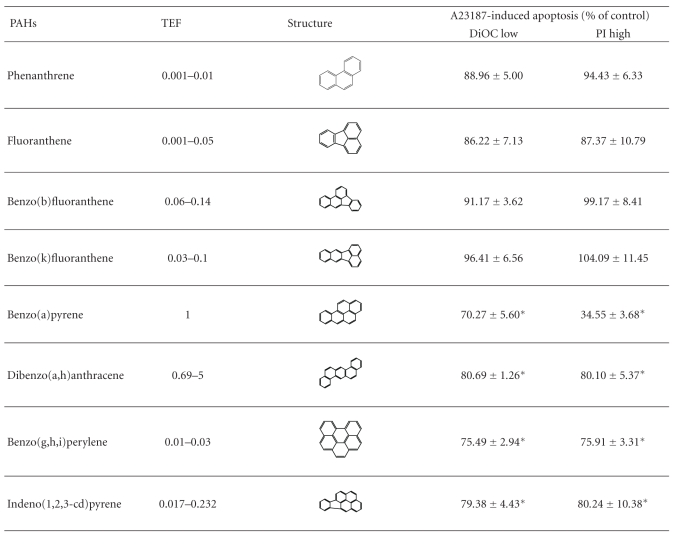

## Abbreviations

2,4-DDE: 2,4-decadienal
8-OHdG: 8-hydroxy-2′-desoxyguanosine
A23187: Calcium ionophore (calcimycin)
AFSSET: French Agency of Environmental and Occupational Health Safety
AhR: Aryl hydrocarbon receptor
AIF: Apoptosis-inducing factor
ANT: Adenine nucleotide translocator
Apaf-1: Apoptosis protease activating factor 1
ARE: Antioxidant response elements
As: Arsenic
BaP: Benzo(a)pyrene
BPDE: anti-7,8-dihydrodiol-9,10-epoxy-benzo(a)pyrene4
CAD: Caspase-activated DNase
Cd: Cadmium
CFCs: Chlorofluorocarbons
CH_4_: Methane
CO: Carbon monoxide
Co: Cobalt
CO_2:_: Carbon dioxide
COF: Cooking oil fumes
COPD: Chronic obstructive pulmonary disease
Cr: Chromium
CsA: Cyclosporine A
Cu: Copper
DEP: Diesel exhaust particles
DIDS: 4,4-diisothiocyanatostilbene-2,2′disulfonic acid
DiOC: 3, 3 dihexyloxacarbocyanine iodide
DISC: Death-inducing signalling complex
DNP: 2,4-dinitrophenol
EGFR: Epidermal growth factor receptor
EndoG: Endonuclease G
EPA: Environmental Protection Agency
FAD: Flavin adenine dinucleotide
FADD: Fas-associated protein with death domain
FP: Fine particles
GSH: L-*γ*-glutamyl-L-cysteinylglycine
GSK3*β*: Glycogen synthase kinase 3*β*
GST: Glutathione S-transferase
H_2_O_2_: Hydrogen peroxide
H_2_S: Hydrogen sulfide
Hg: Mercury
HK II: Hexokinase type II
HO^∙^: Hydroxyl radical
HO-1: Heme oxygenase-1
IAP1: Inhibitors of apoptosis
IARC: International Agency for Research on Cancer
IM: Mitochondrial inner membrane
IMS: Mitochondrial intermembrane space
Keap1: Kelch-like ECH-associated protein
MMP: Mitochondrial membranes permeabilization
mtDNA: Mitochondrial DNA
N_2_O: Nitrous oxide
NAAQS: National Ambient Air Quality Standards
NAC: N-acetylcysteine
Ni: Nickel
NO: Nitrogen monoxide
NO_2_: Nitrogen dioxide
NOx: Nitrogen oxides
NP: Nanoparticles
NQO1: NADPH : quinone oxidoreductase
Nrf2: Nuclear factor (erythroid-derived 2)-like 2
O_2_^∙−^: Superoxide anion
O_3_: Ozone
OM: Mitochondrial outer membrane
OXPHOS: Oxidative phosphorylations
PAHs: Polycyclic aromatic hydrocarbons
PARP-1: Poly (ADP-ribose) polymerase 1
Pb: Lead
PCBs: Polychlorinated biphenyls
PCDDs: Polychlorinated dibenzo-p-dioxins
PCDFs: Polychlorinated dibenzofurans
PI: Propidium iodide
PM: Particulate matter
ppb: parts per billion
ppm: Parts per million
PTP: Permeability transition pore complex
ROS: Reactive oxygen species
Sb: Antimony
Se: Selenium
SO_2_: Sulfur dioxide
SOD: Mn-superoxide dismutase
TCDD: 2,3,7,8-tetrachlorodibenzo-*p*-dioxin
TEF: Toxic equivalency factor
TNFR: Tumour necrosis factor receptor
TRADD: TNF-receptor-associated death domain protein
TSPO: Translocator protein
UFP: Ultrafine particles
V: Vanadium
VDAC: Voltage-dependent anion channel
VOC: Volatile organic compounds
XIAP: X-linked inhibitor of apoptosis protein
Zn: Zinc
ΔΨm: Mitochondrial transmembrane potential.
